# Solid-Phase Synthesis of *N*-Substituted Glycine Oligomers (α-Peptoids) and Derivatives

**DOI:** 10.3390/molecules15085282

**Published:** 2010-08-04

**Authors:** Adrian S. Culf, Rodney J. Ouellette

**Affiliations:** 1 Atlantic Cancer Research Institute, 35 Providence Street, Moncton, NB. EIC 8X3, Canada; 2 Department of Chemistry and Biochemistry, Mount Allison University, 63C York Street, Sackville, NB. E4L 1G8, Canada

**Keywords:** *N*-substituted polyglycine oligomer, peptoid, solid phase synthesis, synthetic methods

## Abstract

Peptoids (*N*-substituted polyglycines and extended peptoids with variant backbone amino-acid monomer units) are oligomeric synthetic polymers that are becoming a valuable molecular tool in the biosciences. Of particular interest are their applications to the exploration of peptoid secondary structures and drug design. Major advantages of peptoids as research and pharmaceutical tools include the ease and economy of synthesis, highly variable backbone and side-chain chemistry possibilities. At the same time, peptoids have been demonstrated as highly active in biological systems while resistant to proteolytic decay. This review with 227 references considers the solid-phase synthetic aspects of peptoid preparation and utilization up to 2010 from the instigation, by R. N. Zuckermann *et al.*, of peptoid chemistry in 1992.

## 1. Introduction

**Figure 1:**
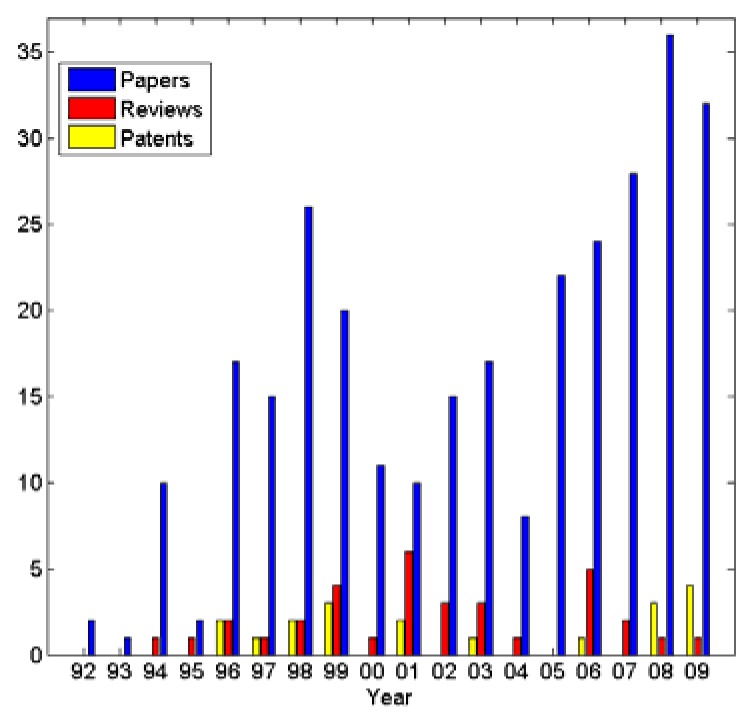
Primary research articles, reviews and patents on NSG’s published from 1992 to 2010.

*N*-Substituted glycine oligomers (NSG), otherwise referred to as α-peptoids, are a readily accessible class of synthetic, non-natural peptide mimic of modular design into which a plethora of structural elements can be readily incorporated. The first NSG reports came from Zuckermann *et al.* in 1992 [1,2]. Since then, the number of reports has been steadily increasing although still coming from a relatively small number of research groups (Figure 1). NSG’s were originally anticipated as a source of lead structure development in the pharmaceutical industry through the preparation of combinatorial libraries of short oligomers [3,4,5]. The initially preferred NSG oligomeric length was a trimer. However, since then the length has extended to 48-mers [6]; 50-mers for homo-oligomers with short linear side-chains; 60-mers *via* chemical ligation of 15-mers [7,8] and even 150-mers by bio-ligation using the cysteine protease, clostripain [9]. Further work on NSG’s has underscored the considerable untapped potential for NSG’s in medicinal chemistry and as molecular biological tools [4,10,11,12,13,14,15,16,17,18,19,20,21,22,23,24] with applications currently extending to nanostructured materials, catalysis and sensors [25,26,27,28,29,30,31,32,33,34,35,36,37,38,39,40 and see Addendum, page 41].

**Figure 2:**
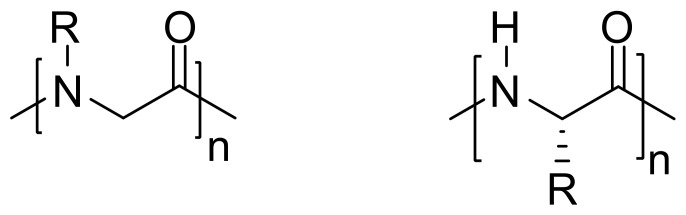
Structure comparison of an α-peptoid and an α-peptide.

In the biological sciences, one of the major applications of NSG’s is in the analysis of protein-protein interactions. Protein-protein interactions are important in the cellular context and the study of these interfaces is needed for fundamental research in medicine and the bio-chemical sciences, protein capture and purification, diagnostics, *etc.* However, direct application of proteins and peptides have some severe limitations as medicinal entities as they are typically degraded by proteolytic enzymes and possess poor cell membrane permeability. NSG’s are structural isomers of peptides. However, in NSG’s the pendant side chain extends from an imino-nitrogen, instead of the α-carbon, leading to an achiral, flexible oligomeric backbone devoid of hydrogen bond donors (Figure 2).

**Figure 3:**
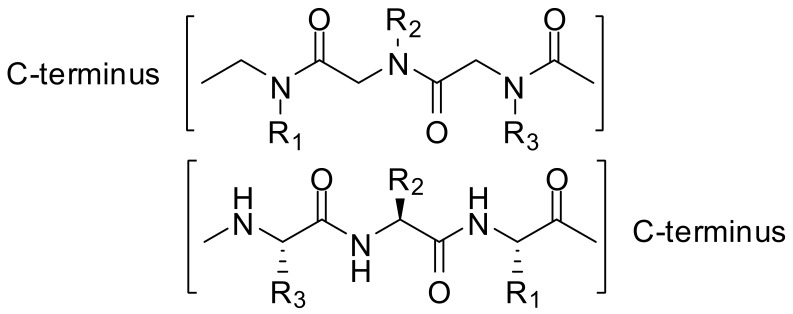
Alignment of retro-α-peptoid (top) and α-peptide sequences.

Thus, when compared to α-peptides, NSG’s have distinct secondary structures (e.g., helices) characterized by steric and electronic interactions that are stable over a wider range of solvent, ionic and thermal conditions [41]. Further, the NSG backbone is not a substrate for commonly encountered proteases which leads to backbone proteolytic stability. In addition, NSG’s can be more hydrophobic and they possess superior cellular permeability [3,4,5,11,19,20,21]. Still, there is primary sequence alignment of carbonyl groups and side-chains between α-peptides and α-peptoids when countercurrent oligomer direction is correlated (Figure 3). In general, NSG’s present a platform for the study of protein interactions beyond those approachable by small molecules defined by Lipinski’s rules and α-peptides.

Recent reviews concerning NSG’s have focused on structure-function relationships and applications [12,16,26,27]. This work provides a comprehensive review of the solid-phase synthesis of *N*-substituted glycine oligomers (α-peptoids) for the period of its inception in 1992 to April 2010. Literature was searched using the American Chemical Society SciFinder Scholar CAS on-line database using the search term “peptoid” with limiters of “Journal”, “Letter” and “Patent” in the English language. Most of these data have been collected into tables for convenient accessibility and critical comparison of the reader. The intention is that the tables are self-explanatory. Only a few topics will be raised in the body of this review. Patents have not been included here, nor have peptoid/peptide hybrids. 

The main table, **Table 1S** (Supplementary Materials), Homo α-Peptoid Oligomer Synthetic Parameters contains full details of experimental protocols used for the solid-phase synthesis of NSG’s, including entries for solid phase-type, reaction scale, amine submonomer predominating, acylation and displacement (amination) chemistries, solvent use, instrumentation, yields and purities recorded for the given NSG chain length together with any distinguishing comments. It was found to be expedient to gather and present the synthesis parameters by research group. The full table with 82 unique synthetic entries is available in the Supplementary Materials accompanying this review. A heavily abbreviated version of this table (**Table 1**) is included in the text and gives a representative appreciation of the salient points for the different reported approaches for the submonomer method of NSG synthesis.

**Table 2** provides details of the solid phases, surfaces and linker chemistry used to immobilise the NSG oligomer during synthetic procedures. A comprehensive inventory of amine submonomers used to date in NSG synthesis is provided in **Table 3a.** The table is sub-divided into **3aa**: aniline, **3ab**: benzyl, **3ac**: benzyl chiral, **3ad**: phenethyl, **3ae**: heteroaromatic, **3af**: miscellaneous aromatic, **3ag**: acyclic alkyl, **3ah**: functionalized acyclic alkyl, **3ai**: cyclic alkyl, **3aj**: amino acids, **3ak**: glycosylamine sub-monomers and **3al**: amino acid monomers. 

The ordering within each table is by the number of substituents, length of main carbon chain or ring size and atomic number of substituent or function other than the prerequisite amine (e.g., _9_F>_8_O>_7_N>_6_C). The most popular amine submonomers (*i.e.* those with the most literature appearances, usually more than five or six) have been collected into **Table 3b**.

Table 4 assembles the automated, robotic, manual, microwave and unique equipment that has been applied for NSG solid phase synthesis.

Table 5 records, in alphabetical order, the methodologies employed in the characterization and study of NSG’s.

Table 6 provides a directory of molecules that have been appended to backbone or side-chain of NSG’s. Further descriptions for this fascinating aspect of NSG application is, unfortunately, beyond the scope of this review. 

Table 7 catalogues the cyclic NSG’s presented in the literature with brief details of the chemistry pertaining to cyclization and the type of cyclization illustrated (e.g*.,* head-to-tail). As most of these protocols are for solution-borne NSG’s they are not dealt with further.

Table 8 lists the main purposes to which NSG’s have been pressed. 

Table 9 is an appreciation of alternate peptoids or related back bone structure oligomers. 

**Table 10** is a comprehensive registry of the chemical formulations used to pry NSG’s from solid phase supports. As in **Table 1S**, the listings are clustered by research group. Reference numbering will skip to these tables before returning to the text at the next section.

## 2. NSG Synthesis Methods

**Scheme 1:**
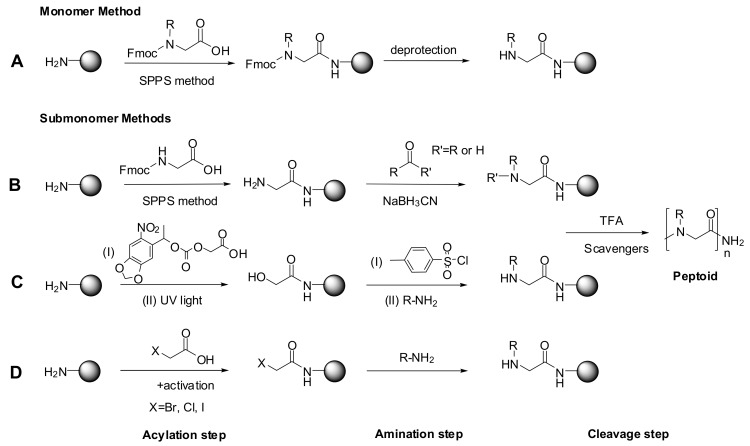
General monomer and submonomer synthetic routes to NSG’s.

There are presently four methods for the synthesis of NSG’s (**Scheme 1**). The first to appear and subsequently called the monomer approach (**Scheme 1A**) has a direct analogy with Fmoc SPPS (Fluorenylmethyloxycarbonyl Solid Phase Peptide Synthesis). Here, previously prepared *N*-Fmoc, *N*-substituted glycine monomers are sequentially coupled creating oligomers [1,29,30,89,90,91,92,98,155,181,214] (**Table 3al**). In the other three tactics, termed submonomer methods, the acyl and amine functions of an amino acid monomeric unit are derived from two sequential chemical reactions of acylation and displacement or amination. One of the submonomer strategies, related to the monomer approach utilizes on-resin reductive amination of glycine monomer (**Scheme 1B**) [215,223,224]. Routes **A** and **B** require the use of more expensive protected glycine monomers and coupling reagents, although there is the benefit of real-time coupling analysis from the UV absorbent Fmoc protecting group fragment, dibenzofulvene. This allows the potential for real-time synthesis remediation [98]. A more recent and very exciting technology as a submonomer strategy for high throughout synthesis is the light-directed route of Kodadek *et al.* (**Scheme 1C**) [53,54]. The use of digital photolithography is a highly attractive path to the development of diagnostics. 

The most common submonomer method is detailed in this review (**Scheme 1D**). In this method, acylation adds an activated carboxylic acid derivative onto a receptive amine to generate a (tertiary) amide bond [2,3,4,5,11]. Typically monobromo- or monochloroacetic acid is used, although the symmetric monobromoacetic acid anhydride [88,109], 2,4-dinitrophenylmonobromoacetate [99,102,103,109] for SPOT synthesis on cellulose membranes as well as the N-hydroxysuccinimide (NHS) ester of monochloroacetic acid [212] have been similarly employed. Subsequent displacement of a halide (most typically bromide) by an amine (typically primary although a secondary amine can be used at the *N*-terminus) produces a secondary amine that is then subject to subsequent acylation thereby propagating the NSG oligomer (**Scheme 1D**). Typical cycle times for NSG oligomer synthesis are of the order of 150-180 minutes for the completion of one monomeric residue addition at room temperature [55]. Elevation of reaction temperature can significantly reduce the cycle time with, as an example, microwave-assisted peptoid synthetic methodology offering cycle times of approximately 5-10 minutes (**Table 4**) [19,22,42,43,44,45,46,47,48,70,85,86,89,90,91,108,142]. 

The scheme represents one monomer addition for the four types of NSG synthesis. Fmoc=fluorenylmethyleneoxycarbonyl, SPPS = solid phase peptide synthesis.

The following sections will follow the NSG synthesis steps as outlined in **Scheme 1**: Synthesis Instrumentation; Solid Phase Support; Acylation; Amination; Solvents; Deprotection; Analysis Methods and Structural Elaboration of Peptoids.

## 3. Synthesis Instrumentation

A wide variety of platforms have been put to use for NSG synthesis, including customized automated peptide synthesizers; Robotic workstations; Microwave synthesizers; Houghten tea-bags to contain pools of resin [70,73]; sonicator and manual equipment such as fritted syringes or chromatography columns (Listed in **Table 1S** and collated in **Table 4,** references therein).

## 4. Solid Phase Support

Polystyrene (PS) and polystyrene-poly(ethylene glycol) block co-polymer (PS-PEG) beads with various linker chemistries, magnetic beads, cellulose membranes, modified glass (microarray) and titanium dioxide surfaces have been utilized for the synthesis or display of NSG’s (**Table 2**). The vast majority of reported syntheses have employed polystyrene beads functionalized with the Rink amide linker leading to C-terminal NSG amides analogous to natural peptide amides. Resultant peptoid amides will have one residual positive charge at the *N*-terminal amine, whereas a peptoid acid will be zwitterionic in aqueous solution with charges at both terminii.

It has been noted that the PS-PEG polymer, TentaGel (Rapp Polymere, Germany), much preferred for its dual compatibility with NSG synthesis conditions and ensuing on-resin biochemical assay development [52], is however sensitive to rapid changes in solvent polarity resulting in bead cracking upon a direct solvent change to water from dichloromethane [62]. It was found that a gradual change in solvent polarity was tolerated with the sequence of dichloromethane to 1:1 (v/v) dichloromethane-methanol to methanol to water maintaining the structural integrity of this solid phase support [62]. The recent introduction of magnetic beads is a ploy to eliminate the need for the re-synthesis of hits in order to harvest a hit from biochemical screening [40,216] using split-pool combinatorial libraries.

Another linker chemistry in evidence for NSG synthesis is 2-chlorotrityl chloride [57,110]. This sterically challenging moiety assists in the suppression of diketopiperazine (DKP) formation at the peptoid dimer juncture. This linker also yields a C-terminal carboxylic acid. Another facet of this linker is facile cleavage, using the benign 20% (v/v) hexafluoroisopropanol in dichloromethane reagent (instead of the standard trifluoroacetic acid-based protocols), enabling subsequent C-terminal modifications such as cyclization [57] or conjugation (see **Table 6**). In contrast, cleavage of a NSG dimer from Wang/HMP resin provides quantitative yields of DKP [110]. Further details on Cleavage and Deprotection can be found in a following section of this review. C-terminal α-amino acids have been exploited for some enabling applications. Cysteine has been applied for subsequent conjugation to the fluorophore, fluorescein [47] following cleavage from Knorr amide resin. The thiol function, generated from cysteinamine 2-chlorotrityl resin has been used for chemical ligation of NSG 15-mers to themselves [7,8]. The same authors made C-terminal aldehyde from Sasrin resin coupled with 2,2-dimethyl-1,3-dioxolane-4-methaneamine [7,8]. Methionine has allowed cyanogen bromide mediated cleavage from Tentagel resin [42] and a C-terminal D-serine-glycine spacer enabled complete Edman degradative NSG sequencing on-resin [50]. 

C-terminal NSG secondary amides have been prepared from MAMP resin (**Table 2**) by the displacement of a chloride from the linker in an initial amination step by amine submonomer prior to the first acylation step by acyl chlorides [107]. Aldehyde functionalized resins have been used to the same effect using an initial reductive amination with amine submonomer and NaCNBH_3_ in a DMF/MeOH/AcOH solvent system [71,72,84,217] with reaction monitoring by the Vazquez test [72]. In a solution synthesis, Blackwell *et al.* produced C-terminal methyl ester, dimethylamide and piperidinamide of *N*-substituted, *N*-acetylglycine monomers [127]. A *p*-gunanidinophenol ester was prepared from a NSG 4- and 5-mer as a protease-mediated ligation substrate in another solution synthesis [9].

An atypical solid support is cellulose membrane employed in SPOT synthesis [99,102,103,109]. These continuous surfaces are used for the preparation of combinatorial libraries in a position addressable manner. Reported is chemically derivatized Whatman 40 cellulose, a type of ashless filter paper, with Rink amide [102,103] and the photolabile 4-[4-(1-Fmoc-aminoethyl)-2-methoxy-5-nitrophenoxyl]butanoic acid [103,109] as linkers. More common among surfaces is glass of the familiar microarray format, with chemical derivatization of amine [53,54], alkyne [93] and maleimide [22,112,113,114] for chemical ligation of NSG’s. Titanium dioxide has also been utilized as a format for the display of NSG’s in investigations of anti-fouling [78]. 

## 5. Acylation

**Figure 4:**
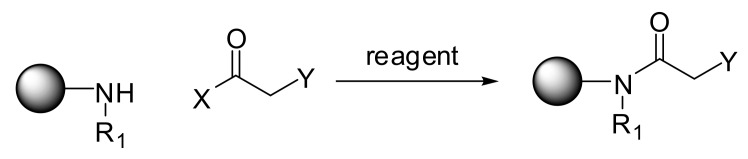
Acylation reaction in NSG submonomer synthesis.

Acylation is the first reaction in the submonomer cycle adding the glycine skeleton to the NSG oligomer (**Scheme 1**). In general, acylation is effected with monobromoacetic acid and the liquid diisopropylcarbodiimide (DIC) reagent (**Figure 4**). The latter is added neat or in DMF solution (see **Table 1S**). A convenient 3.2 M solution is prepared from a 1:1 DIC:DMF (v/v) mixture [3].

Typically, 20 molar equivalents (range 5–200 eq.) of monobromoacetic acid (concentration range 0.4–2.0 M in DMF) are added in a reaction spanning 30 s for microwave assistance to 30 minutes at room temperature (ranges up to 16 hours) (**Table 1 and Table 1S**). Other solvents used are DMF/DCM mixtures [73,74], DCM [72,95], NMP [88,102,103] for monobromo- or monochloroacetic acid or monobromoacetic acid anhydride [88] and DCM [75,76] or THF [107] for monochloroacetyl chloride. Zuckermann *et al.* identified an optimal molar ratio of 0.93:1 (DIC:monobromoacetic acid) [7,8]. Acid activation with DIC has been performed separately from the solid phase support by Albericio *et al.* in order to ensure addition of the formed acid anhydride only with the dehydration urea byproduct being filtered away from a dichloromethane solution [72]. High yields for the acylation reaction have been assured by reaction monitoring using Kaiser [72], deClercq [71,72], bromophenol blue [104] or chloranil [73] tests. 

A slight elevation of temperature to 35 ºC [7,8,62,63,67] or 37 ºC [55] or the assistance of microwaves [55,70,85,86,192] (both monomode and multimode, see **Table 4**) has proven to be beneficial to NSG purity and yield, probably by subjugating NSG secondary structure and its influence on reaction site accessibility. Reaction time and monobromoacetic acid concentration has also been optimized with concomitant increases in yield. Zuckermann *et al.* observed a 50% jump in stepwise yield for acylation by *decreasing* monobromoacetic acid concentration from 1.2 M to 0.4 M and *decreasing* reaction time from 40 minutes to 5 minutes [64]. Similar gains have been discovered by Blackwell *et al.* [86] and Messeguer *et al.* [70].

Although monobromoacetic acid, activated with DIC, is the standard bifunctional acylation/amination synthon in NSG preparation, there exists the dual acylation and alkylation reactivity of this reactant to consider. The selectivity for monobromoacetic acid/DIC is approximately 1000 times in favour of acylation [4,64]. However, in the presence of unprotected heterocyclic aromatic nitrogens or phenols, cumulative alkylation occurs. Monochloroacetic acid was introduced to avoid this, taking advantage of the 40-fold difference in leaving ability between bromide and chloride [64,107]. For a series of NSG 5-mers containing two heterocyclic aromatic amine submonomers at positions 2 and 4 (eleven amines in total that included anilines, pyridines, imidazoles, pyrazine and quinoline) the observed purity improved from a range of 10–87% for monobromoacetic acid to a range of 78–95% for monochloroacetic acid. Yields were similarly improved, being 43–93% for monobromoacetic acid compared to a range of 71–92% for monochloroacetic acid (**Tables 3ab, 3ae**). The acylation reagent monochloroacetic acid/DIC has been widely adopted for instances when heterocyclic aromatic amines are encountered [36,61,64,72,82] or simply for added security against unwanted alkylation [71,72,73,74,107]. Alternative forms of acid activation include the use of monochloroacetyl chloride in company with triethylamine to mop up resulting hydrogen chloride byproduct [75,76,107] - 20 molar equivalents were reacted for 90 minutes on an ice bath [75,76]; symmetric monobromoacetic acid anhydride [88,109] elegantly negates the need for activation reagent; 2,4-dinitrophenylmonobromoacetate was developed to allow preferential *N*-acylation in the heavily hydroxylated cellulose environment [99,102,103,109]. Of note is the negative effect of *N*^1^-hydroxy-benzotriazole (HOBt) on yield, dropping from 75% to 5% upon application of 0.6M HOBt [2]. 

## 6. Amination

**Figure 5:**
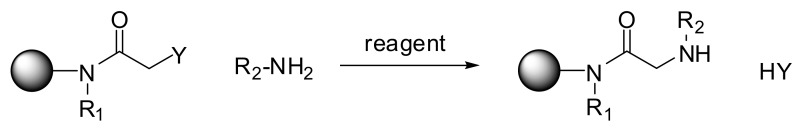
Amination reaction in NSG submonomer synthesis.

Amination, or displacement, is the second and final reaction in the submonomer cycle (Scheme 1). In this step, a primary, or *N*-terminal secondary, amine (called the amine submonomer, **Table 3a, 3b**) displaces a halide anion from the tertiary haloacetamide covalently attached to the solid phase support to complete *N*-substituted glycine monomer addition (Figure 5). This reaction creates the molecular diversity displayed by NSG’s, supported by the many hundreds of available primary amines [4]. In fact, over 1000 amines are said to be commercially available [3], although studies use a small sub-set of this total for any given area of study [3,11]. 230 amine submonomers used in NSG synthesis are listed in **Table 3a**. In general, there is the practice of coded nomenclature for amines used in NSG synthesis. However, there appears to be an absence of rules governing their use and evidence of inconsistent application. Thus, here we avoided them completely, relying instead on the chemical structure and IUPAC chemical nomenclature. Blackwell and co-worker have also noted this confusion [12]. Hence, **Table 3a/3b, Amine Submonomers used for *N*-Substituted Glycine Oligomer Synthesis** does not contain any amine submonomer abbreviated nomenclature, just the chemical structure grouped by chemical class.

Typical reaction conditions are 20 molar equivalents of a 1 M DMF solution of amine submonomer at room temperature for 1-2 hours. Parameter ranges are 5-50 molar equivalents; 0.4–2 M, but not less than 0.5 M [3]; room temperature to 95 ºC; 30 s (microwave) – 16 hours (**Table 1S**). Other solvents are DMSO [2,3,50,51,52,53,54,67,68,71,88,104,106,108], NMP [7,8,60,62,64,79,83,88,107], DCM [83], water/ 0.05% Tween 20 or neat [102,103]. As the recommended amine concentration is 1–2 M, amine solubility can prove to be problematic with the suggestion of sonication in warm water to aid solubilization [3]. The use of a water/0.05% Tween 20 solvent system allowed Wenschuh *et al.* [102] to apply 5 M amine solutions for SPOT synthesis. 

α-Chiral amines are popular as a surrogate for a chiral atom on the NSG back bone [41,58,65,67,79,83,84,85,100,102,125,126,128,136,161,174] (glycine being achiral) thereby inducing helical secondary structure to NSG’s. Zuckermann *et al.* have noted that the α-methyl group is the largest that can be incorporated without incurring losses in yield for primary alkylamines [67]. Interestingly, Lokey and co-worker observed that a primary butylamine fragment was necessary to obtain good yields with amination of 3- or 4-bromomethylbenzoic acids in their work on solid phase synthesis of extended peptoids [207]. Less nucleophilic amines (e.g*.,* nitrobenzylamines) and aryl amines require extended reaction times at room temperature although the application of microwave irradiation and attendant higher temperatures have been most valuable. Kirshenbaum *et al.* [57] used standard conditions (1.2 M anilines in DMF, 20 eq., room temperature) but simply extended the reaction time from 1-2 hours to 16 hours. Fukase *et al.* [104] similarly reacted 15 equivalents of *o*-phenylenediamine at room temperature for 3 hours. Blackwell *et al.* [85,86] pioneered the use of multimode microwave irradiation to facilitate the incorporation of nitrated and fluorinated benzylamines. The increase in product purity for NSG 5-mers was considerable going from a range of 11–92% at room temperature to 56–93% at 95 ºC for 90 seconds with microwaves. One 9-mer gave a comparison of 50% purity at room temperature *versus* 69% with the microwave protocol [86]. The observation of “pale pink oils” resulting from nitroaromatic peptoids [85] may signify the formation of some Meisenheimer complexes during the synthesis.

During the displacement reaction, the HBr byproduct is neutralized by an extra molar equivalent of amine submonomer. In order to eliminate the waste of valuable amine, sacrificial tertiary amine (TEA) has been used such that the amine submonomer can be employed at a lower 5 equivalent [71,72,73,74,75,76] or 10 equivalent level [100] typically in DMF or DMSO [71] instead of the more common 20 molar equivalents of amine submonomer (see **Table 1S**). As some amine submonomers are only available as hydrochloride salts, these have been released by the addition of either DIEA [87] or 0.95 equivalents of 11 M KOH bases [3]. 

As with the haloacetic acids, optimization of reaction conditions has resulted in *decreases* in reaction time. Reaction times of 20 minutes [23,28,33,36,37,38,56,77], 30 minutes [94,95] or 40 minutes [9,58] all at room temperature have been specified. At elevated temperatures; 20 minutes at 35 ºC [62,63] or 60 ºC [89,90,91]; 40 minutes at 35 ºC [7,8,67]; 30 seconds (as 2 × 15 seconds) under multimode microwave irradiation [19,22,40,42,43,44,45,46,47,48] at an undisclosed (obviously the highest) temperature; 60 seconds at 50 ºC [108]; 90 seconds at 90 ºC [70] or 95 ºC [85,86] under monomode microwave irradiation. The most common reaction times are 1-2 hours at room temperature (**Table 1S**). Concentrations of amine submonomer are typically 1-2 M, yet Messeguer *et al.* in contrast have used 0.4 M in DMF [70]. However, they compensated for lower concentration by an attendant increase in the number of molar equivalents from, typically 20, to 50 equivalents. As such, the molar quantity of amine present for amination is essentially unchanged in this microwave assisted account [70]. As the NSG gains in length, Zuckermann *et al.* have gradually increased the reaction time for amination from 20 to 120 minutes for 5- or 10-mers [62] or from 40 to 90 minutes for 15-mers [7,193] all at 35 ºC. 

**Figure 6:**
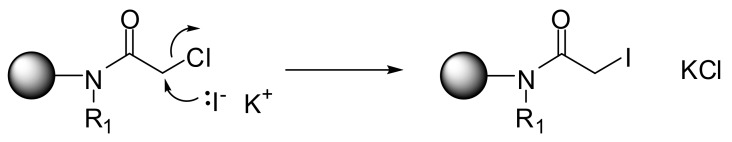
Substitution of Cl for I of greater leaving ability [64,82,107].

Metal iodides (usually NaI or KI) are used to facilitate amination by substituting for chlorine at the resin-bound monochloroacetate [64,82,107] in order to increase leaving ability (**Figure 6**).

*In situ* iodination allows a useful increase in alkylation reactivity without the possibility of cross reactivity with functional groups already introduced on the resin-bound NSG. The use of iodide is particularly effective for valuable NSG’s such as ^13^C-labelling [82] or for amines of reduced nucleophilicity (e.g., anilines) [64]. As in the case of acylation, chemical tests are used to monitor amination reaction progress. These are the bromophenol blue [99], deClercq [71,72], chloranil [72,73] and Beilstein [104] tests. 

## 7. Solvents

Although it has been recently stated that the acylation and amination reactions at the core of NSG oligomer synthesis are not particularly moisture sensitive [11], it had been previously observed that traces of water in anhydrous DMF or DMSO leads to n-1, n-2 NSG oligomers (*i.e. N*-terminal deletion sequences) due to *N*-terminal hydroxyl functions replacing the halogen through hydrolysis thereby terminating chain elongation [55]. As a relatively large volume of solvent is used in any solid phase synthesis for resin washing the use of high purity, anhydrous solvents is an imperative. However, at least three amine submonomers can only be conveniently applied as aqueous solutions – ammonia [120], hydroxylamine [99,102] and methylamine [26,68,120,133] (**Table 3ag**).

Most resin washing protocols in NSG synthesis use DMF (see **Table 1S**). Some deviances from this practice are the DMF/isopropyl alcohol/DCM wash of polystyrene resin with the Rink amide AM RAM linker (**Table 2**) of Messeguer *et al.* [73,74,75,76] and the washing of chemical modified Whatman 40 cellulose with the sequence of DMF(× 4), MeOH, 0.5M NaOH (× 5), MeOH (× 2) and diethyl ether by Wenschuh *et al.* [102].

## 8. Cleavage and Deprotection

At the end of the synthesis, the NSG oligomer remains attached to the resin linker and amine submonomers used during construction may have protecting groups attached to secondary functional groups. It has been advised that aliphatic hydroxyls, carboxylic acids, thiols, amines and heterocycles such as imidazoles and indoles carry protection [3]. As the vast majority of protocols use the acid-labile Rink amide linker (**Table 1a, Table 2**) to yield C-terminal peptoid amide, a similarly acid-labile protecting group regime is adopted, dictating protecting groups such as Boc (*t*-butyloxycarbonyl) for amines, ^t^Bu (*t*-butyl) ester or ether for carboxyl or hydroxyl groups, Trt (trityl) for thiol and heterocyclic amine (eg. imidazole); Pmc (2,2,5,7,8-pentamethylchroman-6-sulfonyl) for the guanidine function of arginine mimics and various silyl ethers (**Table 2**). Chemical interactions between oligomers are largely precluded by the physical isolation of NSG’s from each other on-resin leading to the observation that there is no general requirement for protecting groups [4,218]. 

There exists a range of cleavage cocktails based on TFA (trifluoroacetic acid) (**Table 10**) where the most common system is TFA:TIS:water (95:2.5:2.5 (v/v), TIS is triisopropylsilane) for 1-2 hours at room temperature. Other scavengers are thioanisole [42,88,98]; anisole [52,71]; TES (triethylsilane) [61,65,101]; phenol [130]; *m*-cresol [87] and EDT (ethylenedithiol) [88,98]. Longer deprotection times are used when side-chain protecting groups are present [80,130]. Albericio *et al.* conducted a careful study of cleavage formulations leading to a TFA:DCM:anisole (49:49:2, v/v) system that maximized NSG purity [71]. They state that anisole is the best scavenger for the Boc group. 

The 2-chlorotrityl linker (**Table 2**) offers an alternate deprotection, where solution-borne C-terminal peptoid acid is produced after exposure to DCM:HFIP (80:20, v/v, HFIP is hexafluoroisopropanol) for 30 minutes [30,57]. The resulting side-chain protected NSG’s are valuable for ligation (**Table 6, Table 8**) and cyclization (**Table 7**). 

**Figure 7:**
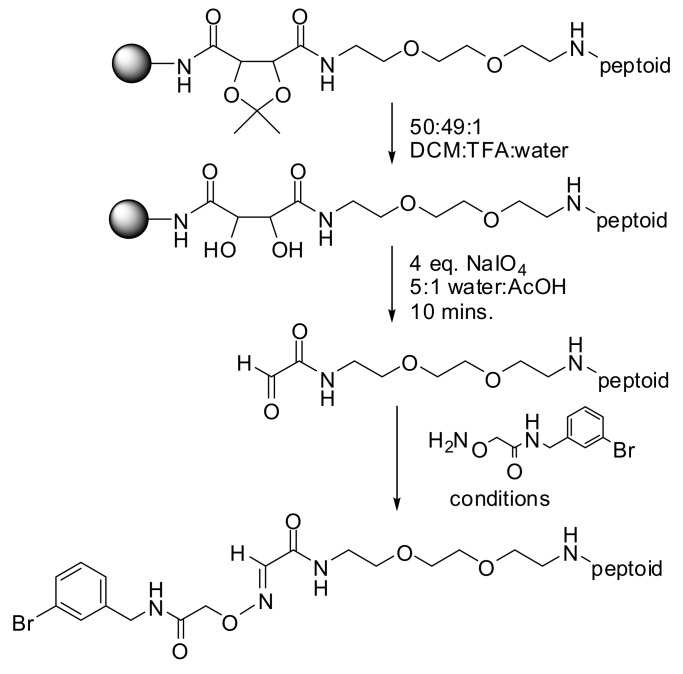
Novel oxidative cleavage used for isotopic tagging [62].

A novel, oxidative deprotection is reported by Zuckermann *et al.* [62], where sequential treatment of the special linker illustrated in **Figure 7** leads to an aldehyde function which is linked to a brominated tag as a Schiff base thereby providing a distinctive probe for mass spectrometric analyses taking advantage of the approximately equal amounts of ^79/81^Br isotopes. 

## 9. Analysis Methods

**Figure 8:**
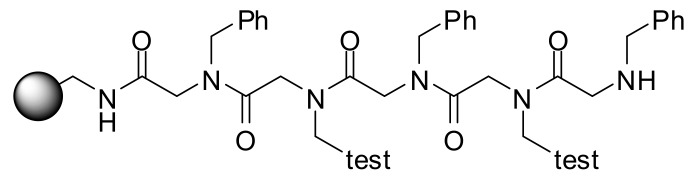
Benzylamine sandwich assay to test for incorporation of new amine submonomers (“test”) [3,10,52,64].

Aside from the chemical tests used to ascertain completion of acylation and amination reactions (see above sections on these two areas), the “benzylamine sandwich assay” is a standardized test of solid phase synthetic procedure effectiveness for the incorporation of a new amine submonomer [3,10,52,64]. A 5-mer with the well-behaved benzylamine interdigitates the test amine submonomer at positions 2 and 4 (**Figure 8**).

A 50% [3] to 85-90% overall isolated yield for the 5-mer [10,52] has been stipulated as thresholds for amine submonomer use in NSG synthesis, so this set point is variable depending upon circumstances [64].

^19^F-NMR of nine aryl fluoride tags has been used to analyse combinatorial libraries [105,106]. Kihlberg *et al.* introduced three aryl fluoride tags in an earlier report [115]. Other analysis methods are enumerated in **Table 5**.

## 10. Structural Elaboration of Peptoids

Peptoid side chains define their physical, chemical and biological properties. Thus, post-synthetic modifications of side chains allows for the development of peptoids for specific applications.

### 10.1. Water Solubility

**Figure 9:**
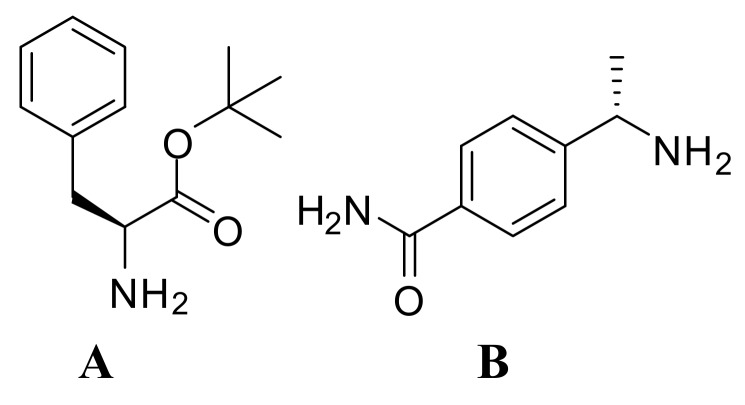
Amine submonomer garnering water solubility in a homo-oligomer (2- to 13-mer) [4,58,60].

Water solubility is a challenge with NSG’s due to their lack of hydrogen bonding donor groups on the backbone and, in general, the scarcity of hydrophilic side chains that have been employed in their synthesis to date. For example, **Table 3b** illustrates that only a few of the most commonly used amine submonomers would be expected to confer water solubility. However, the use of hydroxyl and ether functionalised alkylamines have been usefully employed to this end. Water solubility of helical peptoids has been problematic due to the hydrophobic character of the many bulky, chiral amines that have been observed to induce secondary structure. A typical example would be the α-methylbenzylamines (**Table 3ac, Table 3b**). Kirshenbaum and co-worker [58] developed a helix-forming chiral α-methylbenzylamine NSG using the monomer shown in **Figure 9A** to form water-soluble anionic NSG’s. It was earlier found that chiral-substituted carboxamides imparted an increase in water solubility [4] which was also used in a more recent publication [60], wherein the preparation of a homo-septamer displaying a solubility of 2 mg/mL was detailed, **Figure 9B, Table 3ac**.

**Figure 10:**
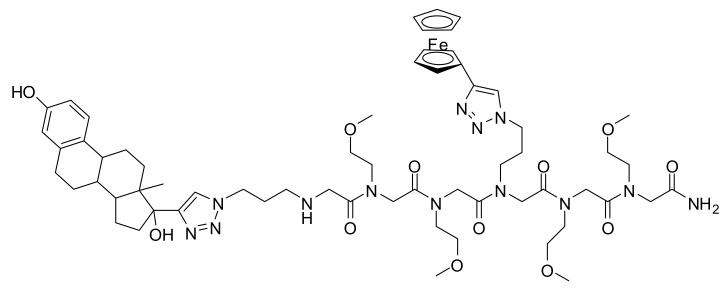
Water soluble peptoid electrochemical biosensor [37].

Kirshenbaum *et al.* also produced a water-soluble electrochemical bio-sensor harnessing the water solubility of the methoxyethylamine submonomer [37], **Figure 10**.

**Figure 11:**
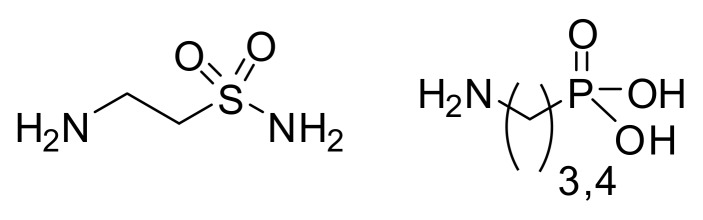
Sulfonamide and phosphoric acid submonomers for water solubility [88,99].

Appella *et al.* produced some novel amine submonomers based on taurine to enhance NSG water solubility [88], **Figure 11, Table 3ah**. Volkmer-Engert also used the sulfonic acid submonomer taurine to the same effect [99].

The cationic 1,4-butadiamine submonomer (lysine mimic) [83] (**Table 3ah**) and the anionic alanine submonomer [41] (**Table 3aj**) have also been used for water solubilization. Kodadek *et al.* have used a number of functionalized amine submonomers in their biomedical studies [52] (**Table 3a**) to ensure water solubility.

### 10.2. Glycosylation

**Figure 12:**
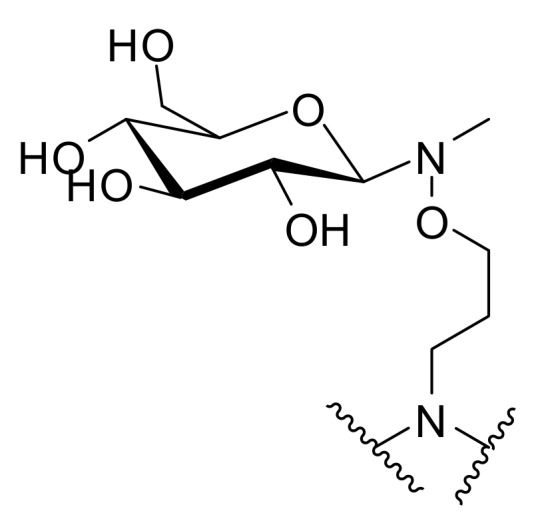
The *N*-methylaminooxypropylamine glycosylation product from Carrasco *et al.* [142,145].

Glycosylation of NSG’s is a preferred conjugative strategy in order to increase bioavailability, absorption and attenuation of *in vivo* clearance (**Table 3ak**) [145]. The most recent report in this area, by Carrasco *et al.*, is an easy to apply method utilizing *N*-methylaminooxypropylamine submonomer and unprotected reducing saccharides (three monoses, two bioses and one triose) in a gentle microwave protocol giving 81-89% yields [142], **Figure 12**.

### 10.3. Side Chain Instability

**Figure 13:**
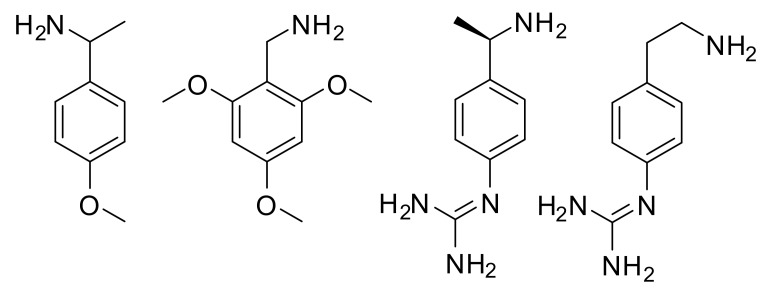
Amine submonomers lost from peptoid during acid cleavage from solid phase support [4,60,93,128].

Some amine submonomers are chemically unstable at the acidic deprotection/cleavage stage of synthesis, usually being lost from the NSG. These include *p*-methoxy α-methylbenzylamine, 2,4,6-trimethoxybenzylamine [4,128] (presumably any electron-rich benzylamine would be a likely suspect), *p*-guanidino α-methylbenzylamine [60] and *p*-guanidinophenylethylamine [93] that have been proposed to be eliminated through a proton-catalyzed mechanism [60], **Figure 13**.

**Figure 14:**
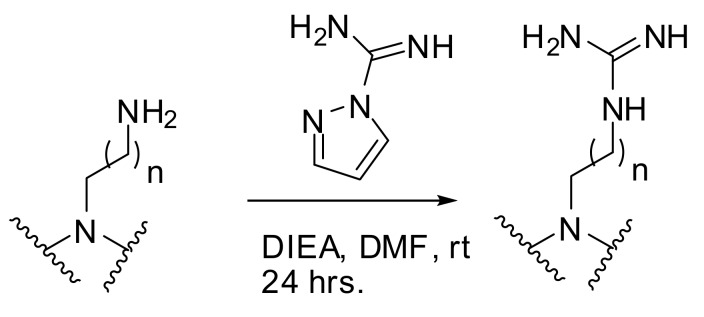
On-resin addition of the guanidine function in NSG synthesis [117,138,155].

However, Disney *et al.* observed that *p*-guanidinophenylethylamine could be retained at the third residue of a tetrameric NSG [93]. The alkylated guanidine group (arginine mimic) has been usefully protected (with Pmc, see **Table 3ah**) and incorporated into NSG’s without difficulty [80,130,140,219]. Another approach has been to add the amidine unit to a side-chain amine already installed onto the NSG using the 1*H*-pyrazole-1-carboxamidine reagent in an on-resin reaction, **Figure 14** [117,138,155]. 

### 10.4. Reductive Dehalogenation

**Figure 15:**
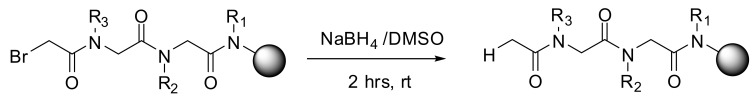
Reductive debromination of *N*-terminal monobromoacetamide [34].

Valuable when using automated instrumentation for NSG synthesis is the reductive debromination of *N*-terminal monobromoacetamide by a 0.25 M solution of sodium borohydride (5 eq.) in DMSO at room temperature for two hours [34] yielding the *N*-terminal acetamide, **Figure 15**.

### 10.5. Isotopic Labelling

**Figure 16:**
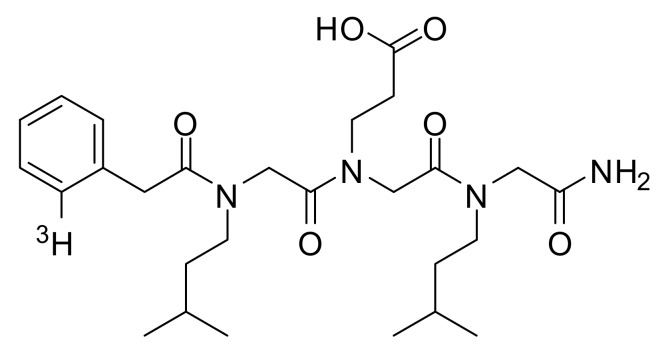
Tritium labelled NSG trimer used in biodistribution studies [225].

Isotopic labelling of NSG’s has produced stable ^13^C and radioactive tritium (^3^H) labelled peptoids. [1,2-^13^C]monobromoacetic acid was incorporated at the C-terminal position of a NSG 9-mer to facilitate two dimensional NMR investigations of a novel threaded loop secondary structure [82]. The same labelled starting material was also used in a mass spectrometric analytical method development [118]. An NSG trimer tritium labelled with [Aryl-^3^H]2-phenylacetic acid was used to follow absorption and disposition in the rat [225], **Figure 16**.

### 10.6. Cyclisation

**Figure 17:**
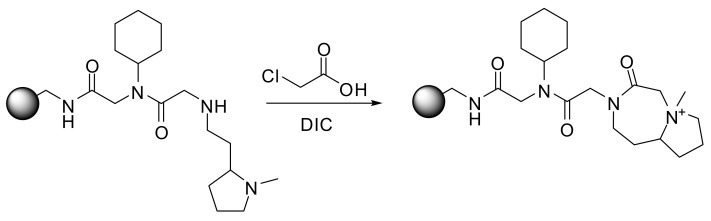
Formation of cyclic ammonium compounds from pendant tertiary amines [73,75].

Some interesting NSG oligomer cyclization reactions can occur. Initial observations were of diketopiperazine (DKP) formation from NSG dimers or cyclic ammonium compounds formed when amine submonomers bearing pendant tertiary amines were present [3]. Messeguer *et al.* made an intensive study of these phenomena in NSG trimer synthesis and showed that where the monomer side chain possessed an unhindered tertiary amine on a two or three carbon chain, subsequent monochloroacetylation would be accompanied by ring closure to a cyclic ammonium compound, **Figure 17**. 

**Figure 18:**
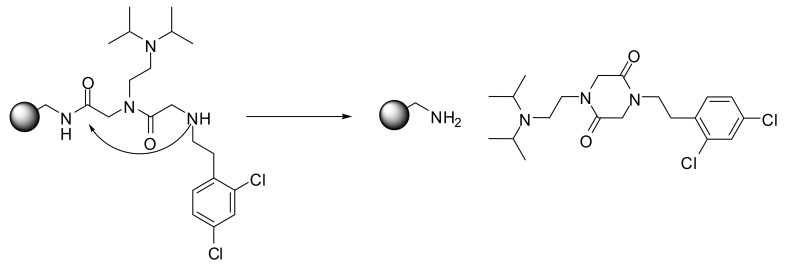
Formation of diketopiperazines from NSG dimers [73,75].

However, if the monomer side chain possessed no amine function or a sterically hindered tertiary amine then the cyclic ammonium outcome was blocked leading to DKP formation [73,75], **Figure 18**. It is advised that NSG synthesis is not halted at the dimer stage as the flexibility of the NSG chain makes DKP formation most likely [3].

### 10.7. Side Chain Elaboration

**Figure 19:**
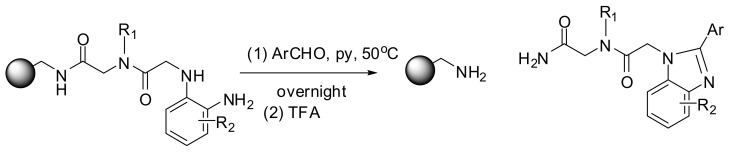
Formation of NSG-benzimidazoles [104].

The installation of *o*-phenylenediamine as an amine submonomer and subsequent condensation with aryl aldehydes in pyridine at 50 ºC overnight led to a range of dimeric NSG-appended benzimidazoles [104], **Figure 19**.

**Figure 20:**
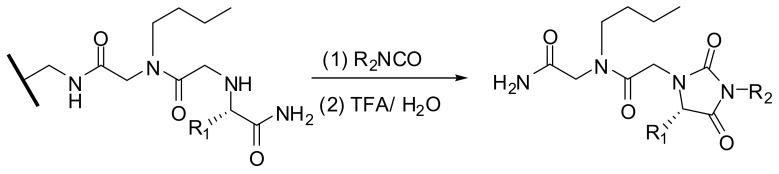
Preparation of NSG-1,3,5-hydantoins [103].

1,3,5-trisubstituted hydantoins have been prepared from NSG dimers, α-amino acid amides or tertiary-butyl esters and isocyanates using concomitant acid-catalyzed ring closure and resin cleavage from a cellulose membrane [103], **Figure 20.**

**Figure 21:**
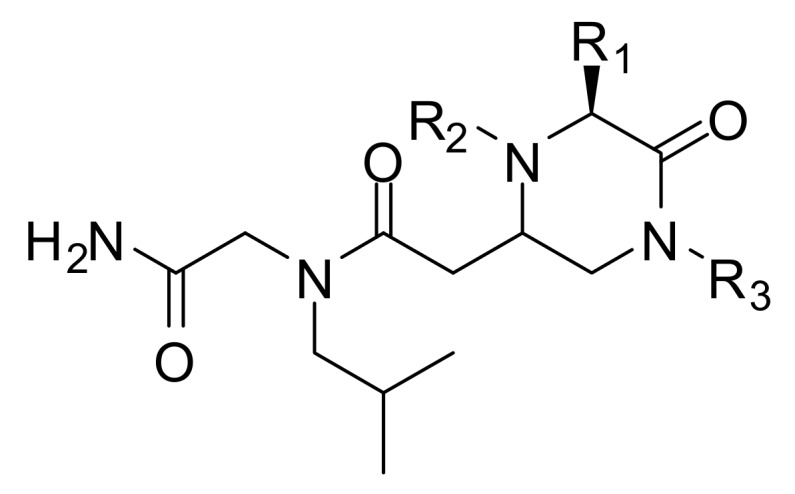
2-Oxopiperazines prepared from NSG dimers [187,220].

2-Oxopiperazines have been synthesized in two different ways. The first was the reaction of an *N*-terminal (E)-4-bromobut-2-enoate NSG dimer with an Fmoc-α-amino acid. Ensuing deprotection and intramolecular aza-Michael cyclization leads to a substituted 2-oxopiperazine appended to the NSG dimer [187], **Table 7**. A later development of the synthetic route to 2-oxopiperazines swapped the amino acid for a peptoid monomer [220], **Figure 21**.

**Figure 22:**
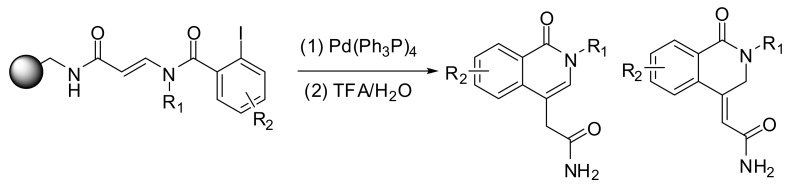
Synthesis of 1(2*H*)-isoquinolinones using NSG synthetic methods [221].

The (E)-4-bromobut-2-enoic acid acylation submonomer was used again in the synthesis of 1(2*H*)-isoquinolinones by an intramolecular Heck reaction of *N*-2-iodobenzamides [221], **Figure 22**. 

**Figure 23:**
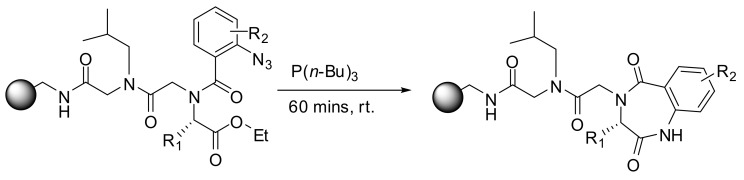
1,4-Benzodiaepines-2,5-diones prepared from NSG dimers [69].

1,4-benzodiazepine-2,5-diones were prepared by an intramolecular aza-Wittig reaction from a NSG dimer *N*-2-azidobenzamide previously aminated by an α-amino acid ester [69], **Figure 23**. 

Naughton and co-worker reported the innovative use of ethylenediaminetetraacetic acid (EDTA) as a core branching unit in peptoid-like syntheses [31]. Any avenue of serious endeavour would be remiss without a little humour and for this we have to thank Kirshenbaum *et al.* with manuscript titles that include: “A new twist on…” [57]; “Peptoids on steroids” [35]; “Fit to be tied” [163]; “Clickity-click” [37] and “Click to fit” [38]! With a healthy total of 14 presentations at the recent 239^th^ ACS National Meeting, March 21-25, 2010 in San Francisco, the future of NSG research and applications appears to be assured.

**Table:** **Glossary** **of Peptoid Terms**

Glossary of Peptoid Terms		
Peptoid	*N*-Substituted glycine oligomer	[1,2]
Affitoid	Synthetic, peptoid-based affinity reagent	†
Ampetoid	Anti-microbial peptoid oligomers	[77,78,222]
Lipitoid	Peptoid-phospholipid conjugate	[129]
Peptomer	Peptide-peptoid hybrid	[162]
Semipeptoid	Cyclic peptoid/amino acid hybrid	[108]

† S. Servoss. www.engr.uark.edu/4122.php.

## Figures and Tables

*N*-substituted glycine *or* α-peptoid oligomer           α-peptide

X = OH (with DIC), Cl (with TEA), NHS, O(CO)CH_2_Y (Y=Cl, Br); Reagent = DIC, TEA

Y = Cl, Br, I; R_2_=alkyl, aryl, heterocyclic; Reagent = base and/or KI

**Table 1:** Abbreviated homo-α-peptoid oligomer synthetic parameters.*

Ref. #	Resin Type	Amine	μW y/n	Acid	Instrument
[42,43,44,45]	TentaGel	benzyl	y	mba	domestic microwave 1kW
[28,33,37,38,70,122]	Rink amide	primary alkyl	n	mba	Illiad 2 robotic workstation, Charybdis Instruments
[70]	Rink amide AM RAM		y	mba	CEM Discover 50mL R.B. flask
[86]	Rink amide	deactivated	y	mba	Milestone MicroSYNTH multimodal microwave
[88]	Rink amide TentaGel	primary alkyl	n	mba anhydride	Pierce fritted PP tube
[102]	Whatman 50	primary alkyl	n	mba	ABIMED AutoSpot Robot
[103]	Cellulose paper			dnp ester	SPOT synthesis
[7,8]	2-Chlorotrityl resin	primary alkyl	n	mba	Auto peptide synthesizer
[72]	BAL resin	amine with 1 eq. TEA	n	mca	PP syringe with PE porous disk
[64]	Rink amide	heterocyclic	n	mca	Auto peptide synthesizer
[75,76]	Rink amide AM RAM	amine with 1 eq. TEA	n	mca chloride	PP syringe with PE porous disk

*Full table of homo α-peptoids oligomer synthetic parameters given in Supplementary Materials. Abbreviations: y = yes, n = no, mba = monobromoacetic acid, mba anhydride = monobromoacetic acid anhydride, mca = monochloroacetic acid, mca chloride = monochloroacetyl chloride, mba dnp ester = 2,4-dinitrophenylmonobromoacetate, TEA = triethylamine; PP = polypropylene, PE = polyethylene, μW = microwave.

**Table 2:** Solid Supports for N-Substituted Glycine Oligomers (α-Peptoid).

Solid Support	Ref. No.
Rink amide MBHA 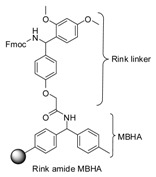	[2,3,7,8,22,28,33,34,35,37,38,46,55,56,57,58,60,61,62,63,64,66,67,68,69,71,72,78,79,80,81,83,86,87,100,101,105,106,108; LL=^23^,^77^]
Rink amide AM RAM 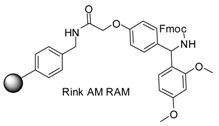	[29,70,73,74,75,76]
Rink amide S RAM 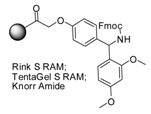	[98]
Knorr amide	[46,47]
TentaGel S RAM/ HL/ MB PS-PEG co-polymer	[62,88; HL: 40; S RAM: 98,104; MB: 42,43,44,45,50,51,52]
Whatman 40 cellulose (Ashless filter paper)	[99,102,103,109]
2-Chlorotrityl chloride polystyrene 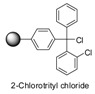 Highly acid labile	[7,8,30,57,63,82,110,111]
NovaSyn TG 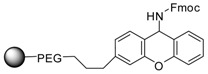 Highly acid labile	[49]
Microarray glass surface 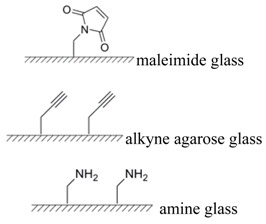	[22,112,113,114] [93] [53,54]
Titanium dioxide (TiO_2_)	[78]
BAL resin 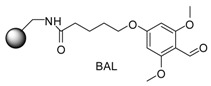	[72,84]
MAMP resin 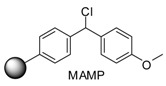	[107]
Sasrin resin 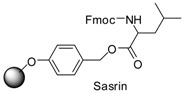	[7,8,63]
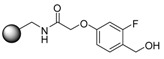 Fluorine linker for gel phase ^19^F NMR	[115]
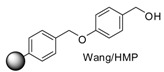 For DKP	[110]

*Abbreviations:* Rink Amide MBHA = 4-(2’,4’-Dimethoxyphenyl-Fmoc-aminomethyl)phenoxy-acetamidonor-leucyl-4-methylbenzhydryl-amine resin; Rink Amide AM = 4-(2’,4’-Dimethoxy-phenyl-Fmoc-aminomethyl)-phenoxyacetamido-norleucylaminomethyl resin; PS = polystyrene; PEG = polyethylene glycol; S = standard; HL = high loading; LL = low loading; MB = macrobead; BAL = backbone amide linker *or* 5-(4-formyl-3,5-dimethoxyphenyloxy)pentanoate-PS; NovaSyn TG = 9-Fmoc-amino-xanthen-3-yloxy TG resin; MAMP = Merrifield, Alpha-methoxyphenyl; HMP = *p*-Benzyloxybenzyl alcohol; DKP = diketopiperazine; 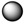 = Polystyrene crosslinked with 1% divinylbenzene.

**Table 3aa:** Amine submonomers used for *N*-substituted glycine oligomer synthesis.

6-atom aromatic, aniline	Ref. No.	6-atom aromatic, aniline	Ref. No.
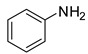 Needs KI additive and MCA	[26,57,64,116,117,118]	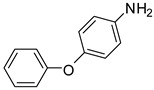	[68,102,120]
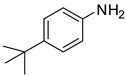	[119]	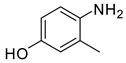	[121]
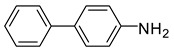	[68,120]	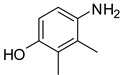	[121]
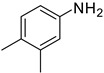	[57]	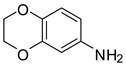	[119,120]
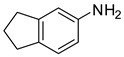	[119,120]	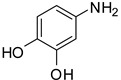	[61]
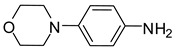	[52]	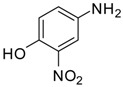	[7,57]
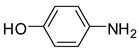	[102]	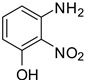	[26]
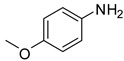	[119]	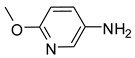	[52,68]

**Table 3ab:** Amine submonomers used for *N*-substituted glycine oligomer synthesis.

6-atom aromatic, benzyl	Ref. No.	6-atom aromatic, benzyl	Ref. No.
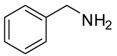	[6,7,26,40,84,86,93,98,99,102,104,110,117,118,120,125,126]	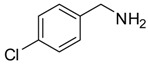	[118,123]
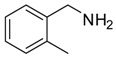	[86]	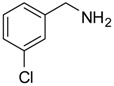	[123]
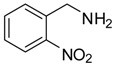	[86]	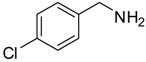	[118,123]
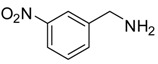	[86]	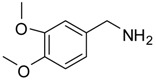	[55,68,118]
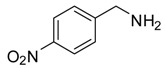	[86]	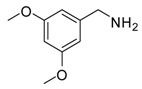	[121]
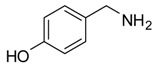	[55,98,99]	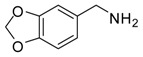	[22,42,50,51,52,55,68,118,120]
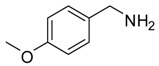	[86,99,102]	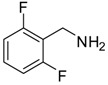	[86]
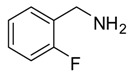	[86]	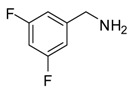	[86]
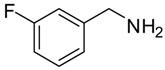	[86]	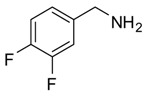	[121]
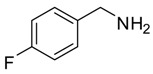	[86,99]	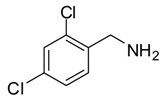	[99]
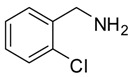	[102]	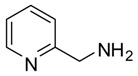	[64,105]
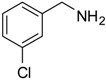	[123]	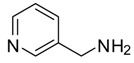	[64,105,118,121]
**6-atom aromatic, benzyl**	**Ref. No.**	**6-atom aromatic, benzyl**	**Ref. No.**
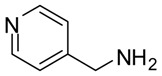	[64]	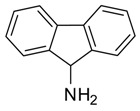	[119]
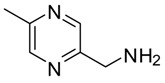	[64]	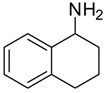	[119,120,123]
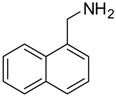	[68]	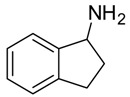	[118,137]
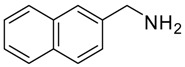	[124]	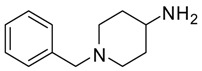	[120]
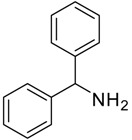	[2,117,118]	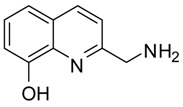	[33]
		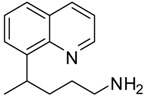	[64]

**Table 3ac:** Amine Submonomers used for *N*-substituted glycine oligomer synthesis.

6-atom aromatic, benzyl chiral	Ref. No.	6-atom aromatic, benzyl chiral	Ref. No.
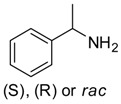	[6,7,22,40,41,46,50,52,55,65,67,80,81,82,83,84,85,86,102,117,125,126]	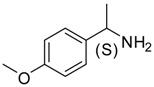	[128]
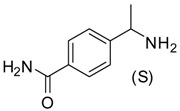	[60]	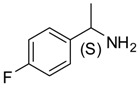	[128]
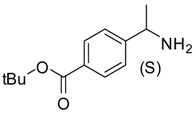	[60]	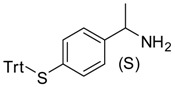	[60]
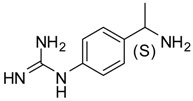	[60]	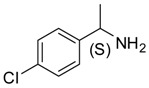	[128]
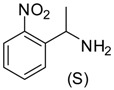	[85]	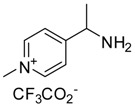	[127]
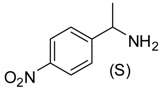	[65,67,85,88,127,128]	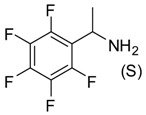	[85,86]
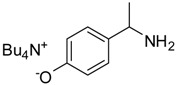	[127]	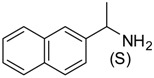	[65,77]

**Table 3ad:** Amine Submonomers used for *N*-substituted glycine oligomer synthesis.

6-atom aromatic, phenethyl	Ref. No.	6-atom aromatic, phenethyl	Ref. No.
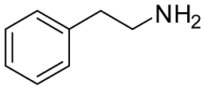	[6,40,42,63,68,73,75,88,117,118,129]	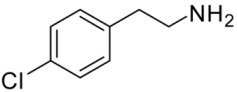	[6,70]
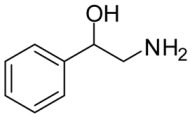	[99,102]	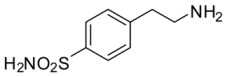	[42,50,51,52,55,73,75,86]
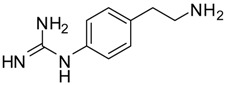	[93]	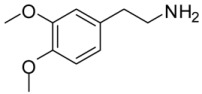	[73,75,99]
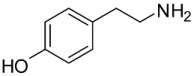	[6,40,46,62,68,99,102,120]	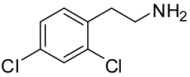	[36,73,74,75,131]
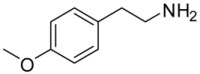	[6,66,70,73,74,75,86,119,120]	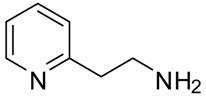	[64,70,73,75,93]
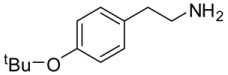 two hours to TFA deprotect	[130]	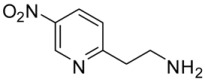	[64]
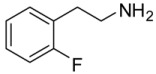	[70,73]	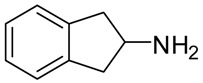	[65]
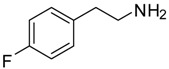	[73,118]	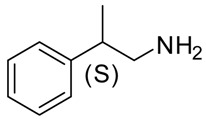	[86,118]

**Table 3ae:** Amine Submonomers used for *N*-substituted glycine oligomer synthesis.

5-atom heteroaromatic	Ref. No.	5-atom heteroaromatic	Ref. No.
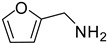	[40,99]	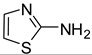	[102]
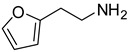	[22,46,52,62,121]	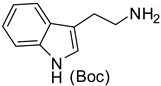	[2,6,40,46,50,51,52,62,64,88,93,99,102,117,124,130]
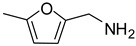	[121]	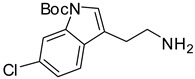	[88]
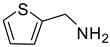	[99,102]	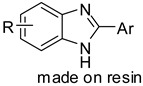	[104]
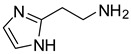	[61,64,99,102]	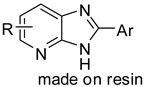	[104]
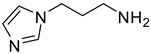	[64,73,75,93,105,119]	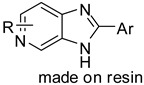	[104]
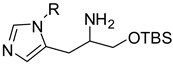	[105]	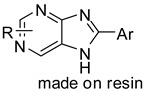	[104]
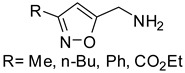	[132]	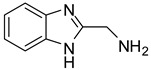	[120]
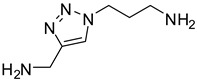	[93]		

**Table 3af:** Amine Submonomers used for *N*-substituted glycine oligomer synthesis.

Aromatic, miscellaneous	Ref. No.	Aromatic, miscellaneous	Ref. No.
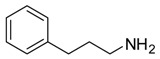	[6,117]	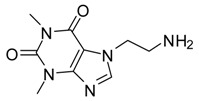	[93]
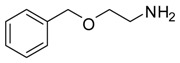	[26,30]	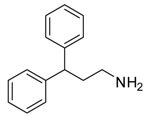	[7,65,68,70,73,74,75,102,118,131]
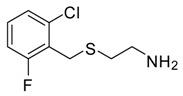	[119,123]	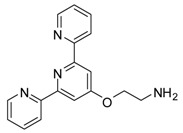	[36]
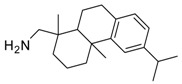	[119,123]	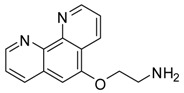	[36]

**Table 3ag:** Amine Submonomers used for *N*-substituted glycine oligomer synthesis.

Alkyl, acyclic	Ref. No.	Alkyl, acyclic	Ref. No.
NH_3_	[120]	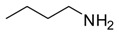	[2,62,68,102,107]
NH_2_-NHBoc	[102]	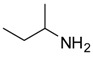	[73,75,102]
NH_2_-OH	[99,102]	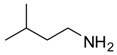	[42,65,73,75,88,129]
Me–NH_2_ (40% in water) (or use sarcosine directly)	[26,68,120,133]	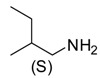	[65,80,81,82,83,135,136]
CF_3_CH_2_NH_2_	[78,119]	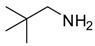	[118]
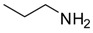	[93,104]	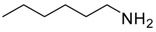	[120]
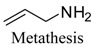	[22,46,52,132,134]	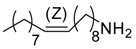	[137]
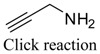	[22,37,38,120,132]	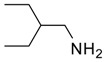	[68]
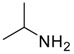	[40,104,126]	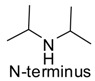	[119]
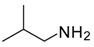	[46,50,51,52,55,68,98,99,102,110,118]	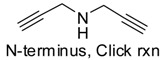	[132]
		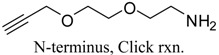	[94]

**Table 3ah:** Amine Submonomers used for *N*-substituted glycine oligomer synthesis.

Alkyl, acyclic with functional group	Alkyl, acyclic	Alkyl, acyclic with functional group	Alkyl, acyclic
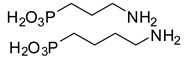	[88]	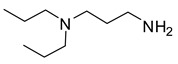	[75]
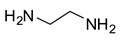	[7,67,119,123,124,129, Boc: 6,66,99,102,138]	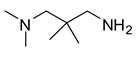	[121]
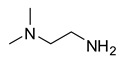	[118]	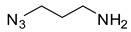 Click reactions	[22,37,38,93]
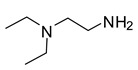	[75]	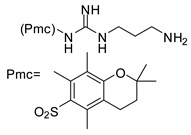	[80,93,117,126,130,140]
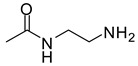	[67,70,73,75,99]	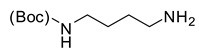	[42,78,80,81,83,99,102,126,138]
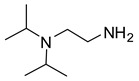	[75]	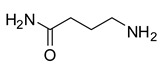	[98]
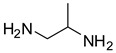	[67]	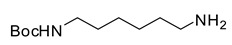	[6,138]
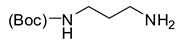	[2,50,52,62,68,119,120 Boc: 6,66,99,102,138]	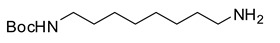	[138]
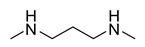 N-terminus	[118]	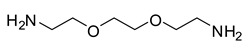	[68,119,120]
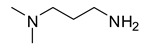	[73,75]	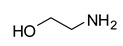	[7,22,42,50,51,52,62,68,88,99,102,119,120]
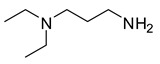	[70,73,75,121]		
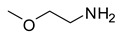	[6,7,26,40,42,51,52,62,63,75,84,86,102,141]	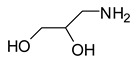	[99,102,119]
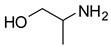	[119]	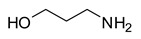	[99,102]
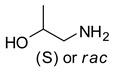	[99,102]	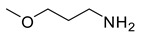	[55]
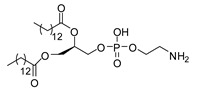	[66,129]	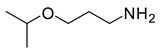	[99,119]
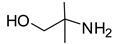	[119]	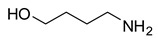	[68]
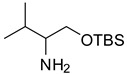	[105]	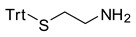	[61,62,119]
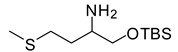	[105]	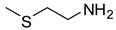	[98]
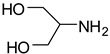	[102]	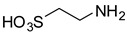	[99]
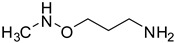	[142]	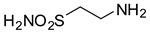	[88]

**Table 3ai:** Amine Submonomers used for *N*-substituted glycine oligomer synthesis.

Alkyl, cyclic	Ref. No.	Alkyl, cyclic	Ref. No.
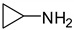	[2,73,75,119]	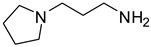	[70]
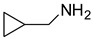	[65,118,120]	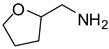	[55,68,70,73,74,86,99,102,118,119,120,121]
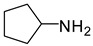	[62,103,115]	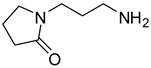	[42,52,73,75,105,118]
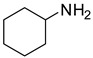	[65,68,75,102]	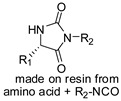	[103]
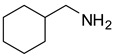	[6,7,84,99,102,117]	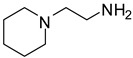	[118]
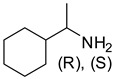	[7,26,65,67,82,83,86,127,136]	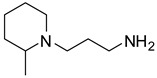	[75]
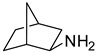	[118]	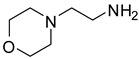	[68,75,118,119]
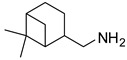	[119]	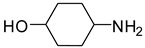	[119]
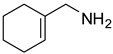	[55]	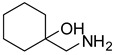	[121]
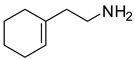	[119]	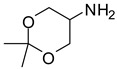	[130]
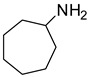	[119,120]	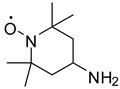	[28,56]
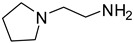	[73,75,118]	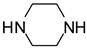 N-terminus or spacer	[118]
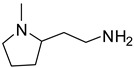	[73,75,105]	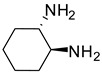	[138]

**Table 3aj:** Amine Submonomers used for *N*-substituted glycine oligomer synthesis.

Amino acids	Ref. No.	Amino acids	Ref. No.
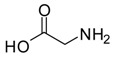	[41,46,62,119,120]	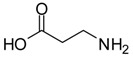	[7,42,68,120]
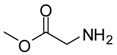	[100]	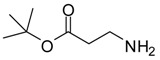	[99,102]
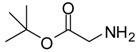	[41,99,102]	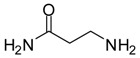	[102]
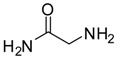	[99,100,102,120]	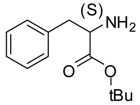	[58]
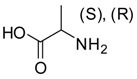	[41,61,67]	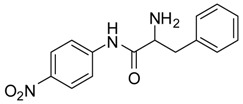	[127]
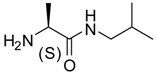	[65]	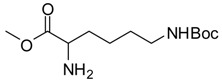	[100]
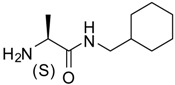	[65]	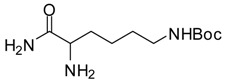	[100]
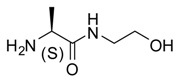	[7,65]	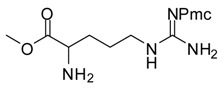	[100]
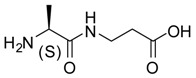	[7,65]	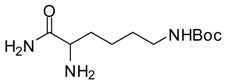	[100]
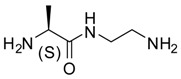	[7,65]	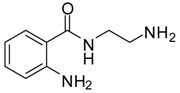	[7]
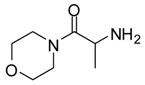	[67]	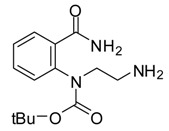	[61]
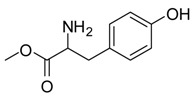	[100]	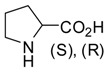	[126]
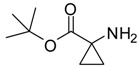	[41]		

**Table 3ak:** Amine Submonomers used for *N*-substituted glycine oligomer synthesis.

Glycosylamines	Ref. No.
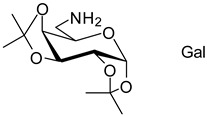	[130,143]
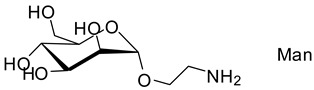	[144]
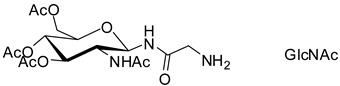	[145,146]
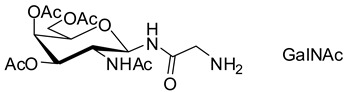	[147]
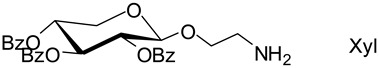	[148,149]
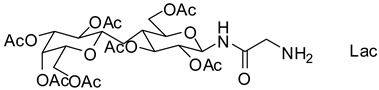	[150]
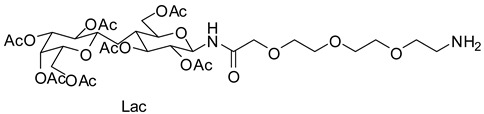	[151]
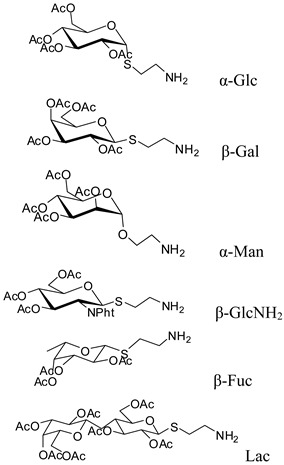	[95]
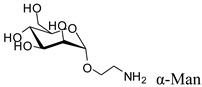	[153]

**Table 3al:** Monomers used for *N*-substituted glycine oligomer synthesis.

Amino acid monomer	Ref. No.	Amino acid monomer	Ref. No.
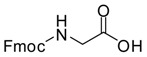	[154]	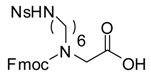	[155]
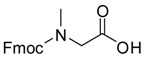	[29,99,102]	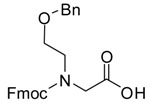	[30]
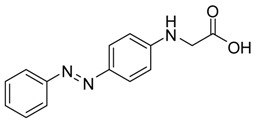	[34]	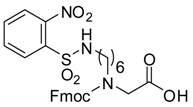	[92]
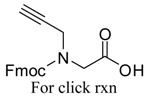	[29]	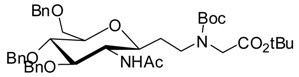	[156]
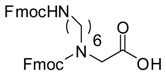	[90]	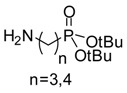	[88]
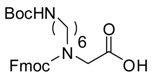	[89,91,92]	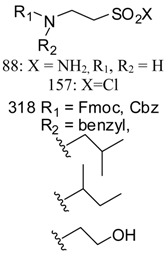	[88,157]
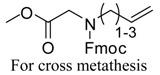	[152]	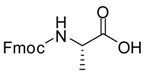	[102]
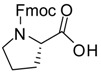	[102]	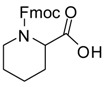	[102]

**Table 3b:** Most Popular Amine Submonomers used for N-substituted glycine oligomer synthesis (full listing of Amine Submonomers given in Table 3a).

Amine	Ref. No.	Amine	Ref. No.
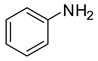 Needs KI additive and MCA	[26,57,64,116,117,118]	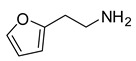	[22,46,52,62,121]
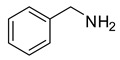	[6,7,26,40,84,86,93,98,99,102,104,110,117,118,120,125,126]	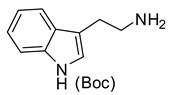	[2,6,40,46,50,51,52,62,64,88,93,99,102,117,124,130]
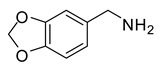	[22,42,50,51,52,55,68,118,120]	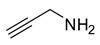 Click reaction	[22,37,38,120,132]
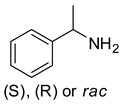	[6,7,22,40,41,46,50,52,55,65,67,80,81,82,83,84,85,86,102,117,125,126]	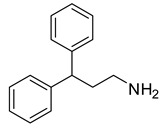	[7,65,68,70,73,74,75,102,118,131]
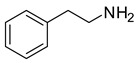	[6,40,42,63,68,73,75,88,117,118,129]	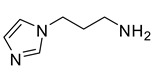	[73,75,93,105,119]
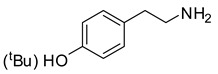	[6,40,46,62,68,99,102,120]	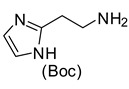	[61,64,99,102]
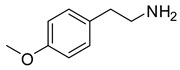	[6,66,70,73,74,75,86,119,120]	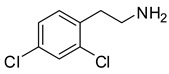	[36,73,74,75,131]
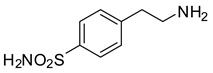	[42,50,51,52,55,73,75,86]		
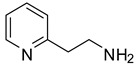	[64,70,73,75,93]	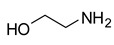	[7,22,42,50,51,52,62,68,88,99,102,119,120]
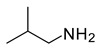	[46,50,51,52,55,68,98,99,102,110,118]	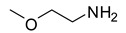	[6,7,26,40,42,51,52,62,63,75,84,86,102,141]
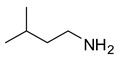	[42,65,73,75,88,129]	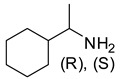	[7,26,65,67,82,83,86,127,136]
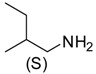	[65,80,81,82,83,135,136]	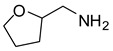	[55,68,70,73,74,86,99,102,118,119,120,121]
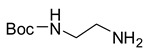	[7,67,119,123,124,129, Boc: 6,66,99,102,138]	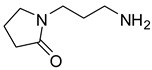	[42,52,73,75,105,118]
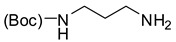	[2,50,52,62,68,119,120 Boc: 6,66,99,102,138]	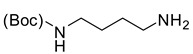	[42,78,80,81,83,99,102,126,138]
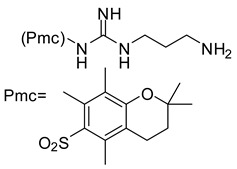	[80,93,117,126,130,140]		

**Table 4:** Synthesis Instrumentation for *N*-Substituted Glycine Oligomer (α-Peptoid) Synthesis.

Synthesis Apparatus	Ref. No.
Automated Peptide Synthesizers	
Rainin 12-channel	[50,51,52]
Symphony (Protein Technologies)	[55,69]
Aapptec Apex 396	[60]
CS Bio 036 Autopeptide synthesizer	[23,77,78]
ABI 433A peptide synthesizer	[80,81,83,98,142]
Microwave Synthesizer	
Domestic, 1kW (Whirlpool) - multimode	[19,22,42,43,44,45,46,47,48,55]
CEM Discover - monomode	[70,108]
Biotage SmithSynthesizer – monomode	[89,90,91]
CEM Mars - multimode	[142]
Milestone MicroSYNTH - multimode	[85,86]
Manual Apparatus	
Innova 4400 Incubator Shaker (New Brunswick Scientific)	[55]
Fritted syringe	[34,35,57]
PP syringe with PE porous disk	[71,72,73,74]
PP fritted tube (Pierce)	[88]
Chromatography column (Bio-Rad poly-prep 0.8x4.0 cm)	[94]
Pipetting onto Whatman 40 paper (SPOT synthesis)	[99,109]
Peptide synthesis vessel (Chemglass, 25mL)	[50,51,52]
Robotic Workstations	
Illiad 2 Robotic Workstation (Charybdis Instruments)	[58]
Robotic Library Synthesizer (Zymark)	[65]
ABIMED Autospot Robot (SPOT synthesizer on Whatman 40 paper)	[102,103]
Other	
Digital photolithography on glass surface (custom instrument)	[53,54]
Sonicator (Branson Bransonic 5210 140W, 47kHz) and Thermolyne Maxi-Mix III stirrer	[93]

**Table 5:** Analysis Methods for *N*-Substituted Glycine Oligomers (α-Peptoids).

Application	Ref. No.
Capillary electrophoresis	[143,158,159]
Combustion analysis / Elemental analysis	[153]
Circular dichroism spectrophotometry	Very common to study secondary structure
Chromatography, Size-exclusion	[65]
Computational Chemistry	
Molecular mechanics	[30,57,67,127,160,161,162,163,164]
Molecular dynamics	[57]
Quantum mechanics	[57,127,165]
Electron microscopy, transmission	[97]
Electron / Paramagnetic spin resonance	[56]
Edman sequencing	[166,167]
Electron microscopy, transmission	[97]
Flash chromatography (9:1 DCM:MeOH)	[98]
Fluorescence, FRET	Very common
High Performance Liquid Chromatography (HPLC)-Analytical and Purification	Vast majority
Infrared (IR)	[97,101,129]
Mass spectrometry (peptoid sequencing)	
Collision Induced Dissociation (CID)	[168,169,170]
Matrix Assisted Laser Desorpton Matrix Ionization time-of-flight (MALDI-TOF)	[167,171]
Isotopic ratio-encoding (^13^C)	[118]
Tandem (MS/MS) MALDI and Surface Enhanced Laser Desorption Ionization (SELDI)	[62]
Nano-electrospray tandem MS with CID	[172]
Microarray	[22,53,54,93,112,113,114]
Nuclear Magnetic Resonance (NMR)	[30,57,67,82,84,106,115,127,128,136,160,163,164,173,174,175]
Ultra-centrifugation, Analytical	[65]
X-ray	[57,127,136,160,164,165,176]

**Table 6:** Peptoid Conjugates.

Peptoid Conjugate Constructs	Ref. No.
Anhydrides	[105]
Azo dye	[34]
Benzimidazoles	[104]
Biotin	[22,144]
Boronic acid	[177]
β-Peptoid	[178]
Chalcones	[179]
DOPA	[141,180]
Ferrocene	[37]
Fluorescent tag	[7,22,47,51,61,89,92,94,138,144,155,181,182]
Glycan clusters	[183]
Hydantoins	[103]
Lipid	[16,25,66(lipitoid),^129^]
Metal complexation: Fe(III), Cu(II)	[30,31,33,36,56,61,105]
Nitroxide radical spin probe	[56]
N-terminal tag for (microarray) crosslinking	[22,63,93,112,113,114]
Oligonucleotide (drag-tag)	[24,158]
Peptide	[141,184]
Polyamide; poly-L-glutamic acid	[45,131]
Purine	[44,78]
Steroid	[19,35,37,48,51]

**Table 7:** Cyclic *N*-Substituted Glycine Oligomers (α-Peptoids).

Cyclopeptoid	Ref. No.
Cyclic poly(N-butylglycine), range of molecular weights NHC ROP of N-butyl,N-carboxylanhydrides 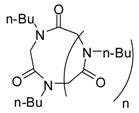	[185]
Cyclic α,β-Alternating Peptoids Solution phase synthesis 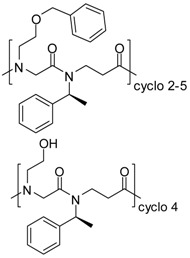	[175]
*N*-Benzyloxyethyl cyclic peptoids 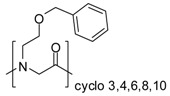 pyBOP(3eq.), DIEA(6eq.), 18 hrs.	[30,160]
Side chain-to-tail cyclic peptoids β-Ala-Cys-β-Ala-C-terminus 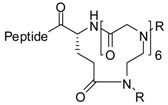 pyBOP (3eq.);HOBt(3eq.);DIEA(10eq.), 5 mins.	[113]
Side chain-to-side chain cyclic peptoids 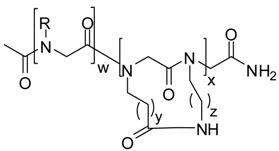 w = 2; x = 3; y = 2 or 4; z = 3 or 5 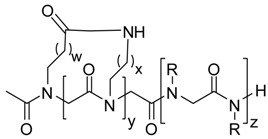 w = 2 or 4; x = 3 or 5; y = 3; z = 3 HATU(3 eq.),DIEA(9eq,), 24 hrs.	[186]
Head-to-tail cyclopeptomer 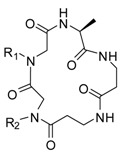 Piperidine(2eq.) from Wang-PS resin. 11 examples.	[162]
Side chain-to-side chain click reaction – intra- and intermolecular 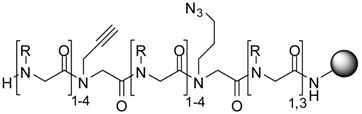 R=Benzyl or (S)-α-methylbenzyl. 12 examples.	[163]
Head-to-tail 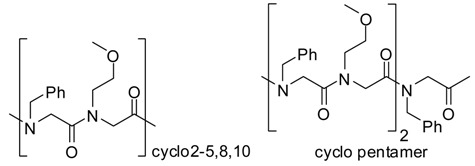 PyBOP(30eq.), DIEA(30eq.), 5 mins. +4 other examples	[164]
Side chain-to-N-conjugate 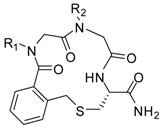 13 examples. 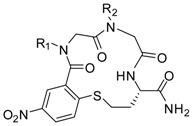 7 examples	[108]
Head-to-tail 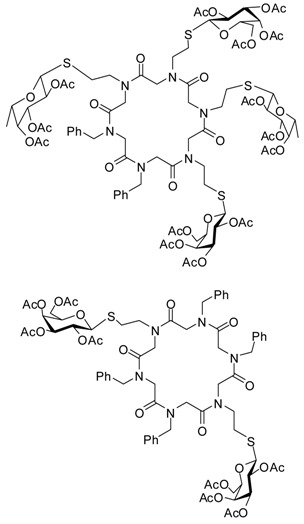 pyBOP(5eq.), HOBt(5eq.), DIEA(19eq.), 12 hrs.	[95]
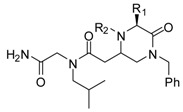 Using (E)-4-bromobut-2-enoic acid for second acylation and α-amino acid for third acylation (R_1_). 6 examples.	[87]
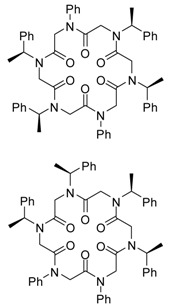	[57]

**NHC=**N-heterocyclic carbene; **ROP**=Ring Opening Polymerization.

**Table 8:** Applications of *N*-substituted Glycine Oligomers (α-Peptoids).

Application	Ref. No.
Anti-cancer	[44,46,47,72,88,131,147,177,179,180,184,188]
Anti-fouling	[23,141]
Anti-fungal	[189]
Anti-microbial (inc. cholera toxin)	[42,74,75,77,83,119,123,135,190,191,192,193]
Anti-viral (mostly HIV)	[100,157,194]
Asymmetric catalyst (model enzyme)	[28,33,36]
Lung surfactant	[25,79,126]
Metal complexation	[36,105]
Alkali	[30]
Cu(II), Co(II)	[33]
Zn(II)	[61]
Muscular dystrophy	[183]
Nanostructures, electrochemical biosensor,	[31,37,195]
Nucleic acid hybridization probe	[24]
Protein Binding	
α-melanotropin (α-MSH)	[196]
Amyloid inhibitor	[97]
Antibody surrogate	[43,102,137]
Chloecystokinin B (CCK-B)	[197]
Clostripain (cysteine protease)	[9]
Concanavalin A (ConA)	[153]
General protein binding	[49]
Glycoprotein P (P-gp) – multidrug resistance reversal	[73]
G-Protein Coupled Receptors (GPCR)	[68,198]
Human Double Minute 2 (HDM2); protein-protein interactions in p53 suppression	[88,179]
Human Melanocortin MC1,3-5R	[117,199]
Neuromedin B	[200]
ORL-1 (Opiod receptor 1)	[201]
Quorum sensing	[162]
Semaphorin 3A	[75,202]
Src Homology Domain (SH3); protein-protein interactions in eukaryotic signal transduction	[121]
Transient Receptor Potential Vanilloid 1 (TRPV1)	[71,73,76]
Trypsin	[70]
Vascular Endothelial Growth Factor Receptor-2 (VEGFR2)	[46,47]
Transcription factor mimic	[45,50,51,203]
Transfection agent	[6,66,90,92,129,155,181]

**Table 9:** Other Types of *N*-Substituted Amino Acid Oligomers (Peptoids).

Other Peptoid	Ref. No.
β-Peptoid, chiral building blocks. β-Peptoid are prepared from β-alanine (3-aminopropanoic acid or 3-bromopropanoic acid)	[204]
α,β-Alternating peptoids- linear and cyclic	[175]
α,β-Alternating peptoids, cationic	[182]
α,β-Alternating peptoids. 8 to 16-mers	[190,205]
β-Peptoids. Chiral (R )- and (S)-1-(phenylethyl)-amine submonomers	[206]
Cyclo-β-Peptoids, 2-6-mers. Further derivatized by click reaction	[165]
Extended peptoids. Using 3- and 4-bromomethylbenzoic acid. 2- to 5-mers. Requires primary amine submonomers with long, straight chains.	[207]
α,β-Alternating peptoids, chiral. Antimicrobial	[191]
β-Peptoid nucleic acid. N^1^-(2-aminoethyl)thymine as amine submonomer. 6-mer.	[208]
β-Peptoids. Chiral (S)-1-(phenylethyl)-amine submonomer. 11-mer.	[209]
Review article on β-peptoids.	[210]
β-Peptoids. Block ligation up to 18-mer. Antimicrobial	[192]
Hydrazino-Azapeptoids. 3-mers. Proteasome inhibitors.	[177]
Aminooxy α-peptoids. 4-mers	[211]
β-Peptoids. 3-mers	[212]
Ureapeptoids. 3-mer. Retains one secondary amide N-H for hydrogen bonding.	[213]

**Table 10:** Cleavage cocktails.

Ref. No.	TFA	DCM	TIS	water	Time	Temp	Comments
	%	%	%	%	mins	oC	
**TK**							
[40,46,47]	95		2.5	2.5	120	rt	
[45,50,51]							
[42]	94		2	2			Plus 2% thioanisole
[48]	95			5	120	rt	
[52]	95			2.5	120	rt	Plus 2.5% anisole
**KK**							
[33,37,57]	95			5	10	rt	hydroxyquinoline
[57]		80			30	rt	Plus 20% HFIP
							2-chlorotrityl resin
[58]	95			5	120	rt	
[19]	95			5	15	rt	
**RNZ**							
[60]	42.5	50	2.5	5	5	rt	
	95		2.5	2.5	10 to 20	rt	t-Bu ester
[23]	95		2.5	2.5	20	rt	
[61]	95			2.5	20	rt	Plus 2.5% TES
							S-trityl deprotect
[61]	95			5	20	rt	
[62]	49	50		1	120-180	rt	
[64]	49	49		2	60	rt	
[65]	30			2	840		plus 67% DCE and 1% TES
[130]	88		2	5	20 - 120	rt	Plus 2% phenol.
							Longer time for
							tBu and Pmc removal
[66]	95			5	20	25	lipitoids
[3,68]	95			5	20	rt	Method Enzymol. Review
[2]	95			5	20	rt	JACS 1992 paper
							1st submonomer paper
**AM / FA**							
[70,73,74,75,76]	60	40		2	30	rt	
[71]	49	49					Plus 2% anisole
							Boc deprotection
							Optimized cleavage cocktail.
[72]	95			5	69	25	
**AB**							
[23,77,80]	95			5	10	rt	60 mins for NArgPMC
[79]	90				15-40	rt	plus scavengers
[81,83]	95		2.5	2.5			
**HB**							
[85,86]	95			5	20-120	rt	20 mins for acid sensitive groups
**DA**							
[87]	95				60		Plus 5% *m-*cresol
							Triazole monomer
[88]	80			12.5	90	rt	Plus 5% EDT and 2.5% thioanisole
**MB**							
[89]	95		2.5	2.5	180		
[90]	90	5	5		120		
**MD**							
[93,94]	60	40		2	60	rt	
**FdeR**							
[96]		80			30		Plus 20% HFIP
							2-chlorotrityl resin
**RMJL**							
[97,98]	95		2.5	2.5	180	rt	
[98]	88		1	4.5			Plus 2% EDT and 4.5% thioanisole
							
S. Brase [155]	95		5		30	rt	
Moos/ Winter [132]	80	20					
T. Rana [100]	95		2.5	2.5			
R. Rocchi [101]	95			2.5	210	rt	Plus 2.5% TES
H, Wenschuh [102,103]	95			5	45	rt or 60	SPOT synthesis on
							cellulose paper
K. Fukase [104]	100				30	rt	
P. A. Wender [138]	95		5				
D.S. Brown [107]	23	75		2	60	rt	

**Notes:** TK=Kodadek group; KK=Kirshenbaum group; RNZ=Zuckermann group; AM / FA = Messegeur and Albericio groups; AB=Barron group; HB=Blackwell group; DA=Appella group; MB=Bradley group; MD=Disney group; FdeR=Riccadris group; RMJL=Liskamp group.

## Addendum

Two important reports have appeared during the review of this manuscript. Zuckermann *et al.* have assembled thin two-dimensional sheets from peptoid 36-mers [226] and Kirshenbaum *et al.* have introduced peptoid synthesis into the undergraduate laboratory with a report of an anti-cancer trimer synthesis for a 4 hour laboratory session [227].
